# A New Unabridged Pronouncing Dictionary of Medicine

**Published:** 1894-03

**Authors:** 


A New Unabridged Pronouncing Dictionary of Medicine, being a voluminous and exhaustive hand-book of Medical and Scientific Terminology, with Phonetic Pronuniciation, Accentuation, Etymology, etc. By John M. Keating, M. D., LL. D., and Henry Hamilton, with the collaboration of J. Chalmers Da Costa, M. D., and Frederick A. Packard, M. D. With an Appendix. Price, cloth, $5.00 net; sheep, $6.00 net. Sold only by subscription. W. B. Saunders, publisher, 913 Walnut Street, Philadelphia, Pa.

